# LSTNet: A Reference-Based Learning Spectral Transformer Network for Spectral Super-Resolution

**DOI:** 10.3390/s22051978

**Published:** 2022-03-03

**Authors:** Debao Yuan, Ling Wu, Huinan Jiang, Bingrui Zhang, Jian Li

**Affiliations:** School of Geoscience and Surveying Engineering, China University of Mining and Technology (Beijing), Beijing 100083, China; sqt2100204089@cumtb.edu.cn (L.W.); zqt1900204082g@cumtb.edu.cn (H.J.); zqt2000204110@cumtb.edu.cn (B.Z.); zqt2100204114@cumtb.edu.cn (J.L.)

**Keywords:** hyperspectral image, spectral super-resolution, reference-based learning, attention network, convolutional neural network

## Abstract

Hyperspectral images (HSIs) are data cubes containing rich spectral information, making them beneficial to many Earth observation missions. However, due to the limitations of the associated imaging systems and their sensors, such as the swath width and revisit period, hyperspectral imagery over a large coverage area cannot be acquired in a short amount of time. Spectral super-resolution (SSR) is a method that involves learning the relationship between a multispectral image (MSI) and an HSI, based on the overlap region, followed by reconstruction of the HSI by making full use of the large swath width of the MSI, thereby improving its coverage. Much research has been conducted recently to address this issue, but most existing methods mainly learn the prior spectral information from training data, lacking constraints on the resulting spectral fidelity. To address this problem, a novel learning spectral transformer network (LSTNet) is proposed in this paper, utilizing a reference-based learning strategy to transfer the spectral structure knowledge of a reference HSI to create a reasonable reconstruction spectrum. More specifically, a spectral transformer module (STM) and a spectral reconstruction module (SRM) are designed, in order to exploit the prior and reference spectral information. Experimental results demonstrate that the proposed method has the ability to produce high-fidelity reconstructed spectra.

## 1. Introduction

A hyperspectral image (HSI) is a data cube recording hundreds of narrow-bandwidth images over a large wavelength range. With the advantage of high spectral resolution, HSIs have many useful applications in fields such as atmosphere monitoring [1], food science [2], agricultural monitoring [3], and medical science [4]. To make full use of the rich spectral information, many HSI processing technologies have been proposed, such as band selection [5], feature extraction [6], image unmixing [7], fine classification [8,9], and object detection [10].

However, due to the limitations of the associated imaging systems, the swath width of an HSI is smaller than that of a multispectral image (MSI), even when they have the same or similar spatial resolution; for example, Gaofen 5 has a 60 km-wide swath, while that of Landsat 8 is 185 km. Due to the orbital revisit period limit, rapid revisiting of the same and surrounding area is difficult to achieve for some satellites. For the above-mentioned reasons, an HSI with a large coverage area is difficult to achieve within a short amount of time.

In the field of remote sensing, researchers have proposed various super-resolution techniques, including hyperspectral image super-resolution (HSI SR) and spectral super-resolution (SSR) [11]. HSI SR refers to reconstructing a high-spatial resolution HSI from an input low-spatial resolution HSI [12]. If a corresponding high-spatial resolution MSI is used as input to the model, HSI SR can be divided into single-image SR and fusion-based SR. However, HSI SR cannot solve the problem of obtaining large HSI coverage in a short time. Compared to the handful of satellite constellations offering HSI, MSIs have a larger swath width and rapid ability to revisit through the use of multiple satellites. If we can make full use of existing MSIs to generate an HSI, the problem relating to the lack of HSI data can be properly addressed.

For this reason, researchers have proposed SSR technology, which aims to reconstruct an HSI with fine spectral resolution from an RGB image or MSI [13]. In contrast to an HSI’s narrow spectral sampling range with hundreds of bands, an RGB image or MSI usually has a wider sampling range and a small number of bands. In general, this reconstruction task is an ill-posed problem, as the solution is not unique. This is mainly because the information contained in an MSI is less than that of an HSI when they have the same or similar spatial resolution, such that there are many possible combinations of HSIs corresponding to a single MSI.

To address this ill-posed problem, many SSR approaches have been proposed, which can be divided into two categories: shallow learning and deep learning [14]. Early researchers mainly used sparse coding or relatively shallow learning methods to explore the prior information of HSIs. However, the limited generalization ability of these methods allows them to perform well only in specific image domains. In recent years, deep learning-based methods have achieved extraordinary success in the computer vision field. Benefiting from the generalization ability of the associated models, deep learning-based methods have also been shown to be impressively effective for SSR [15].

Although the performance of existing SSR methods in learning the mapping functions from MSIs to the corresponding HSIs has been shown to be promising, they mainly depend on the ability of a convolutional neural network (CNN) to learn the spectral prior from the training data. Most CNN-based methods design a wider or deeper network to promote richer contextual information, thereby lacking more constraints on the spectral fidelity. As the input is only a single MSI, the reconstruction result can only rely on the prior spectral information learned by the model, thus potentially falling short of producing more valid spectral structure information.

To address this issue, a novel Learning Spectral Transformer Network (LSTNet) for HSI SSR is proposed in this paper. Specifically, the framework of the proposed method comprises a reference-based super-resolution (RefSR) strategy, in which the spectral structure of a reference HSI can be transferred, through a learning-based approach, in order to create a reasonable reconstruction spectrum. First, in contrast to traditional CNN-based SSR methods, the proposed method couples an MSI with an HSI (Ref-MSI and Ref-HSI, respectively) and utilizes the spectral information to enforce spectral fidelity. More specifically, a spatial attention module is used to compute the spatial relevance between the input MSI and Ref-MSI, and the related spectral structures are transferred from the Ref-HSI to the reconstruction backbone module. Second, multi-level residual-in-residual channel attention is used to reconstruct a backbone module in order to make full use of the global and local residual learning, thereby allowing the information to pass quickly from the bottom to the top level. Furthermore, a channel attention mechanism is used to enhance the channel-wise adaptive learning, through use of which the model can reallocate the weights along the spectral dimension.

The main contributions of the proposed methods are summarized as follows:

1. A novel learning spectral transformer network (LSTNet) for SSR is proposed, which introduces the transformer architecture into the spectral super-resolution problem. More specifically, the LSTNet is composed of a spectral reconstruction module (SRM) and a spectral transformer module (STM), which learn the prior spectral information and reference spectrum.
2. The proposed STM acts as a bridge to transfer the spectral structures learned from the reference image through the attention mechanism. In the STM, a spatial relevance between input MSI and reference MSI was computed to migrate the most relevant spectrum and the spectrum synthesized by the relevance weights, fusing the spectrum from the reference image into an SRM.
3. The proposed SRM learns the prior spectral information of the training data using a channel attention mechanism to adjust the weights between the feature image bands adaptively. Instead of using an average pooling strategy to calculate the feature statistics, dual-channel attention is used to enhance the diversity of features.

## 2. Related Work

In recent years, HSI reconstruction has attracted increasing attention, and numerous methods have been proposed. Approaches to this task can be divided into three strategies. The first involves reconstruction of the spatial resolution, which includes single-image SR [16,17] and fusion-based SR [18]. The purpose is to enhance the spatial resolution of the HSI. The second is spatial–temporal fusion [19], which involves fusing temporally dense and spatially coarse images with temporally sparse and spatially fine images to generate a temporally dense, high-resolution image. The third is enhanced spectral resolution [13,20], which recovers an HSI with finer spectrum from a coarser MSI. In this study, we mainly focus on deep learning-based spectral resolution enhancement. Therefore, we summarize the related work on SSR methods and attention mechanisms in the following.

### 2.1. Spectral Super-Resolution

Over the past few years, an increasing number of methods for SSR have been proposed, and CNN-based methods have achieved especially significant improvements over traditional methods, which often require prior knowledge assumptions to restrict the reconstruction task. Arad et al. [19] have proposed a fast, low-cost method to recover a high-quality HSI directly from an MSI. Their approach uses a hyperspectral prior to create a sparse dictionary of spectral signatures and their corresponding MSI projections. Using the given spectral response function (SRF), a reconstructed HSI can be generated from an MSI through use of a sparse representation. Rang et al. [20] have proposed a radial basis function, using “white balancing” as an intermediate step, for the reconstruction of a spectral reflectance image from a single MSI. This approach also brings the SRF into the model as a known term. Jia et al. [21] have proposed a manifold learning method to recover an HSI from an MSI with a known SRF, with the assumption that the spectral information lies on an intrinsically low-dimensional manifold. However, most of these methods take the SRF as a known prior into the model, making the model highly dependent on this prior information. Furthermore, these traditional methods are pixel-level solving processes, which cannot be used for local and non-local spatial information.

CNN-based SSR has gained increasing attention recently, due to its deep non-linear mapping and spatial information integration capabilities. Xiong et al. [22] first up-sampled MSIs in the spectral dimension through simple interpolation or compressive sensing measurements, and then used an end-to-end CNN-based network to recover the HSI from the up-sampled MSI. Shi et al. [23] have proposed two strategies to reconstruct HSIs from MSIs. One is a deep residual network comprising a number of residual blocks; the other is an architecture that replaces the residual block with a dense block featuring a fusion scheme. Stiebel et al. [24] introduced a modified UNet to learn multi-scale contextual information. Zhang et al. [25] proposed a pixel-aware deep function mixture network, composed of a function mixture block to determine the receptive field size and mapping function in a pixel-wise manner. Li et al. [26] proposed a novel adaptive weighted attention network with multiple dual residual attention blocks, which also utilizes the fact that the MSI can be projected by the reconstructed HSI using the known SRF to further enhance the constraints for more accurate reconstruction. Li et al. [27] proposed a deep hybrid 2D–3D CNN based on dual second-order attention with an SRF prior. Hang et al. [28] proposed an intrinsic property guided end-to-end network consisting of a decomposition sub-network and a self-supervised sub-network. He et al. [29] proposed a universal SSR network based on physical optimization unfolding, enabling it to handle arbitrary MSIs, including single-resolution and cross-scale MSIs.

The existing SSR methods mentioned above focus on the spectral reconstruction of an HSI using a single MSI as input, lacking further auxiliary spectral information to enhance the reconstruction accuracy of the spectrum. Attention mechanisms provide the possibility of using additional reference images to further promote spectral reconstruction.

### 2.2. Attention Mechanisms

In recent years, attention mechanisms have been shown to play a superior role in computer vision tasks, including image classification [25,28,30,31,32], semantic segmentation [26], objection detection [33,34], and image super-resolution [26,29]. We introduce the work related to attention mechanisms from two aspects: channel attention and spatial attention.

**Channel Attention.** Hu et al. [30] proposed a squeeze-and-excitation network that introduces a novel channel attention mechanism to significantly improve image classification accuracy. Although this network allows for reassigning the weights of channel features, it only employs one feature statistic through global average pooling, thus lacking further excavation of features. Zhang et al. [32] proposed a deep residual single-image super-resolution network which uses a channel attention mechanism. Zhang et al. [31] proposed a context-encoding module to capture the semantic context of senses and selectively channel class-dependent feature maps. Yang et al. [33] proposed a channel normalization layer to reduce the associated computational complexity and the number of parameters. Dai et al. [34] proposed a second-order attention network to explore the feature correlations of intermediate layers for image super-resolution. Qin et al. [35] proposed a novel multispectral channel attention involving the pre-processing of a channel attention mechanism in the frequency domain.

**Spatial Attention.** Liu et al. [36] proposed a non-local operation to compute the response as a weighted sum of the features at all positions. Hu et al. [37] presented an object relation module for object detection tasks that processes a set of objects by computing the reasoned relations between them simultaneously, instead of using individual recognition. Chen et al. [38] proposed a graph-based global reasoning network that captures global relations between relation-aware features. Carion et al. [39] proposed a novel method that views object detection as a direct set prediction, from which the use of many hand-designed components and anchor generation are removed. This method interprets the relations between objects and the global image context to predict the final output directly. Yang et al. [40] proposed a texture transformer network for image super-resolution, in which the spatial attention between a low-resolution image and a reference low-resolution image is calculated to further transfer the texture information.

Although current SSR algorithms utilize attention mechanisms to enhance the learning ability of the models, these methods lack further optimization of the spectral structure. To address this problem, we propose a spectral transformer network to enable the network to search for and learn spectral structure information.

## 3. Proposed Method

**Figure 1:**
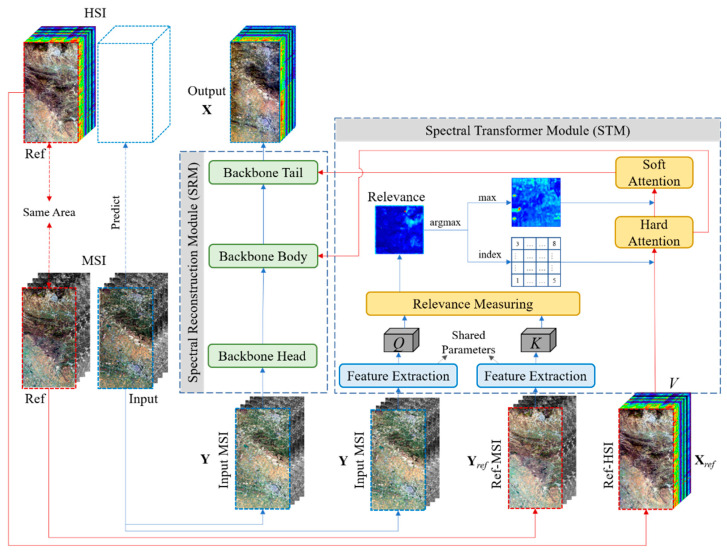
Network architecture of the proposed LSTNet. The input MSI is the image to be processed. The ref-MSI and ref-HSI are a paired MSI and HSI, used as reference images. *Q* and *K* are the features extracted from the input MSI and ref-MSI, denoting query and key, where the used feature extraction sub-networks are shared parameters. *V* denotes the spectral values transformed from the ref-HSI. Relevance denotes the spatial attention relevance between the input MSI and ref-MSI.

The challenge we faced was how to take advantage of the spectral structure of the extra reference image and the prior training data. This drove us to design a network that could exploit both prior and reasonable spectral migration. The overall architecture of the proposed LSTNet for HSI SSR is illustrated in Figure 1. It consists mainly of two modules, the Spectral Transformer Module (STM) and the Spectral Reconstruction Module (SRM), the detailed structures of which we present below.

### 3.1. Degradation Module

Let $X\in {\mathbb{R}}^{W\times H\times C}$ represent the desired target HSI, where *W* and *H* are the width and height and *C* is the number of HSI bands, and $Y\in {\mathbb{R}}^{W\times H\times c}$ represent the input MSI, which has the same spatial size as **X** and where *c* is the number of MSI bands. The paired MSI–HSI reference is denoted by $Y_{ref}\in {\mathbb{R}}^{W\times H\times c}$ and $X_{ref}\in {\mathbb{R}}^{W\times H\times C}$. For convenience, we call the reference MSI and HSI “ref-MSI” and “ref-HSI”, respectively. The relationship between the target **X** and observation **Y** can be formulated as:

(1) Y=RX+E,

where $R\in {\mathbb{R}}^{C\times c}$ denotes the SRF, representing the transformation matrix from the hyperspectral sensor to the multispectral sensor, and **E** is the residual.

The SSR task aims to recover the target **X** from the observation **Y**. According to (1), the spectral reconstruction (i.e., obtaining **X** from **Y**) is an inverse task, which is an ill-posed problem. Consequently, we want the recovery task to have more constraints and extra information in order to improve the reconstruction results. The spectral transformer module is key to transforming the spectral information from the extra reference image.

### 3.2. Spectral Transformer Module

As shown in Figure 1, the STM takes **Y**, **Y***_ref_*, and **X***_ref_* as inputs and exports soft attention and hard attention into the SRM module. First, the feature extraction sub-networks (as discussed below) are used to generate the corresponding features *Q* and *K* from **Y** and **Y***_ref_*, in which the feature extraction sub-networks involve shared parameters. As the features *Q* and *K* are extracted from different images using the same feature extraction network, these features contain more useful spatial texture information to further compute the spatial relevance between **Y** and **Y***_ref_*. The process of feature extraction can be expressed as:

(2) $$Q=f_{FE}(Y),\;$$

(3) $$K=f_{FE}(Y_{ref}),\;$$

where *f_FE_*() denotes the feature extractor. The basic elements of the attention mechanism are *Q* (query), *K* (key), and *V* (value). As we wish to transform the spectral structure from the reference HSI, we use ref-HSI directly as *V*.

Relevance measurement aims to compute the spatial relevance between **Y** and **Y***_ref_* by estimating the spatial similarity between *Q* and *K*. Since both are multispectral images, the spatial similarity facilitates the migration of spectral information on the ref-HSI. Following the setting of attention [34,41], to calculate the relevance, *Q* and *K* are unfolded into patches to make use of the spatial information of the local neighborhood. The patches are denoted as $q_{i}\in {\mathbb{R}}^{\left(n\times p\times p)\times (W\times H\right)}$ and $k_{j}\in {\mathbb{R}}^{\left(n\times p\times p)\times (W\times H\right)}$, where *i* and *j* denote the *i*th and *j*th patches, *n* denotes the number of features of *Q* and *K*, and *p* denotes the patch size. Then, the patches must be normalized, such that the relevance *r_i,j_* can be calculated by using the inner product between each *q_i_* and *k_j_*:

(4) $$r_{i,j}=\frac{q_{i}}{\left\|q_{i}\right\|}\cdot \frac{k_{j}}{\left\|k_{j}\right\|},$$

where ‖‖ denotes the normalization process.

Hard attention in the STM describes the most relevant spectrum in *V* for each *q_i_*, which is the raw spectral information migrated directly from the ref-HSI. Here, we use an argmax function to calculate the index location of the maximum value for each relevance *r_i,j_*, along the dimension of *k_j_*. The resulting indices are the position of the most relevant spectrum in *V*. The hard attention mechanism can be presented as:

(5) $$h_{i}=\underset{j}{\arg\max}r_{i,j},$$

where *h_i_* denotes the index location of each relevant maximum. All of the index locations for the most relevant spectra are formed as a location map, **H**, in order to select the spectrum directly from the ref-HSI *V*. The selected relevance spectrum is further transferred into the spectral reconstruction module in order to help recover **X**. The transformed spectrum features from the hard attention can be formulated as:

(6) $$F_{h}=V_{H}.$$

Soft attention in the STM denotes the spectrum synthesized by the relevance weights. In contrast to the hard attention, where we use an argmax function to determine the index location of the maximum value, soft attention uses the maximum relevance to soften the transformed spectrum from the hard attention. The maximum relevance can be formulated as:

(7) $$s_{i}=\underset{j}{\max}r_{i,j},$$

where *s_i_* denotes the maximum relevance coefficient. Then, all of the coefficients can be formed into a coefficient map **S**, and the synthesized spectrum features can be formulated as:

(8) $$F_{s}=S\cdot F_{h},$$

where **F***_s_* is also a transformation in the spectral reconstruction module.

**Figure 2:**
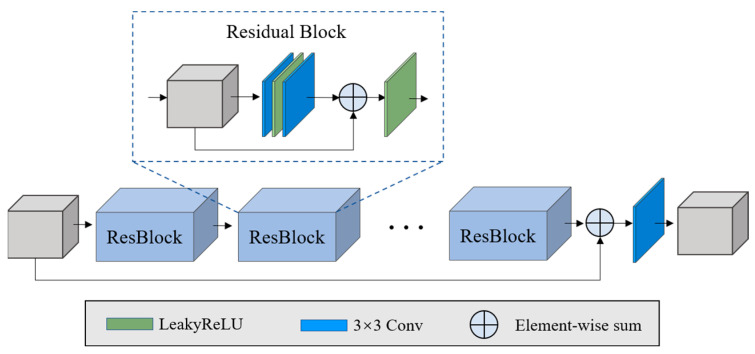
Illustration of the feature extraction sub-network.

We used a classic residual network [42] as the feature extraction sub-network, as shown in Figure 2. Ten residual blocks were stacked and all the strides of the convolutional layer were set to 1 in order to maintain the size of the feature maps.

### 3.3. Spectral Reconstruction Module

**Figure 3:**
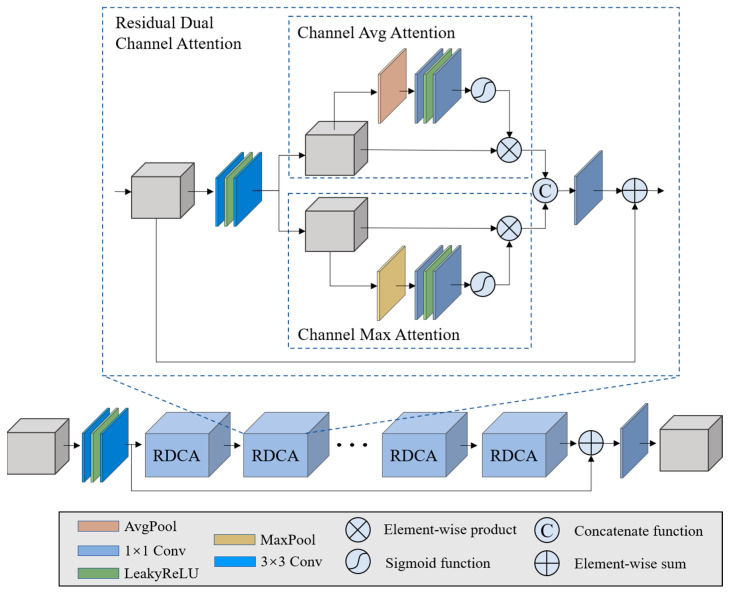
Illustration of the backbone network.

As shown in Figure 1, the SRM module is composed of three backbone sub-networks. The architecture of each backbone sub-network is illustrated in Figure 3. The reason we call them backbone sub-networks is because the role of the STM module is to borrow the spectral information from ref-HSI, while the role of the SRM is to store the mapping priors in the form of the learned parameters in the neural network.

To mitigate the gradient disappearance problem, which can occur in deep networks, a global residual connection was adopted. The overall network was formed by stacking a set of residual dual-channel attention blocks (RDCA) in order to form a deep network for spectral reconstruction. Each RDCA consists of a local residual connection, a set of convolutional layers having 3 × 3 kernel size, and two-channel attention sub-modules. Based on the global and local residual connections, the network allows for the abundant low-frequency features of the input data to be bypassed and the high-frequency features to be sufficiently extracted. In the RDCA, average channel attention and channel max attention were used to exploit the adaptive learning of channel weights. Similar to CBAM [43], we exploited the inter-channel relationships of features through the use of two pooling layers. The average pooling layer calculates the average spatial statistics, and the max-pooling layer calculates distinctive object features. In contrast to CBAM, we further calculate the two-channel reallocated features separately. The two features are then combined through a concatenation function. Therefore, in the backbone network, feature redistribution between channels benefits learning of the prior information of spectral features.

In the SRM, three backbone sub-networks were used to learn the spectral reconstruction process: the backbone head only takes the input MSI as an input to learn the preliminary features, while the backbone body and tail take the transformed hard and soft attention spectra as inputs, along with the features from the previous level, as shown in Figure 1. In summary, the transformed spectral features and spectral priors are combined in the SRM to boost the accuracy of spectral reconstruction.

## 4. Experiments and Results

To verify the performance of the proposed method, we conducted an extensive series of experiments including sensitivity analysis, simulation data experiments, and real data experiments.

### 4.1. Experimental Data Sets and Setting

**Simulation data**. Several widely used HSI data sets were used as simulation data for this experiment. These are the Pavia University data, Pavia Centre data, and University of Houston data.

The Pavia University data were acquired by an ROSIS-3 airborne sensor in 2003. These data have the size of 610 × 340 pixels and consist of 115 bands. Due to the effects of noise and water vapor absorption, 12 bands were removed. The ground sample distance (GSD) of these data was 1.3 m, and the spectral range covers 430 to 840 nm. The Pavia Centre data were also acquired by an ROSIS-3 sensor, with a size of 1096 × 1096 pixels and consisting of 102 bands after removing the water vapor absorption bands.

**Figure 4:**
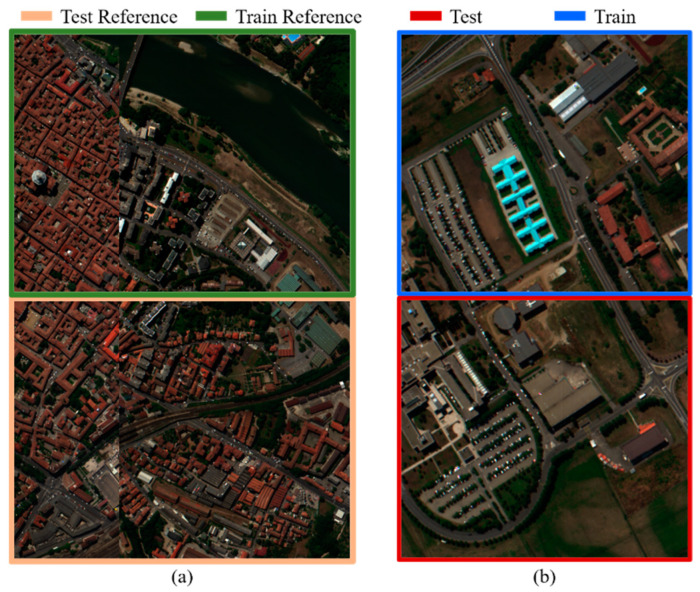
Original Pavia Centre and Pavia University data sets: (**a**) Pavia Centre; (**b**) Pavia University. The border color indicates the purpose of the image.

To test the performance of the spectral transformer, the Pavia Centre data were used as reference imagery and Pavia University data were used as the input imagery. As shown in Figure 4, we divided the images into two parts for training and testing. Each part of the Pavia Centre data consisted of 548 × 1096 pixels, and each part of the Pavia University data consisted of 305 × 340 pixels. To simulate MSI, the blue–SWIR1 SRF of Landsat 8 was used to simulate MSIs from HSIs for all the images.

The University of Houston data were acquired by an ITRES CASI 1500 sensor over the University of Houston campus and its neighborhood on 16 February 2017. The original data have the size of 1202 × 4172 pixels at a 1 m GSD, with 48 bands covering the 380–1050 nm spectral range. These data were provided for the 2018 IEEE GRSS data fusion contest.

**Figure 5:**
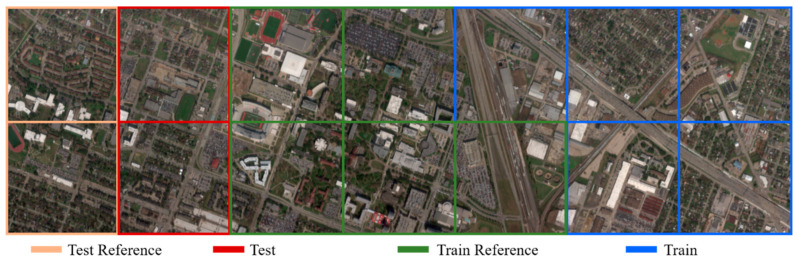
The original University of Houston data set was separated into many pieces, each with size of 601 × 596 pixels. The border color indicates the purpose of the image.

To verify the proposed reference-based transformer, we split these data into several pieces, where the size of each piece was 601 × 596 pixels, as shown in Figure 5. To simulate MSI, the blue–SWIR1 SRF of Landsat 8 was used to simulate MSIs from HSIs for all the pieces.

**Figure 6:**
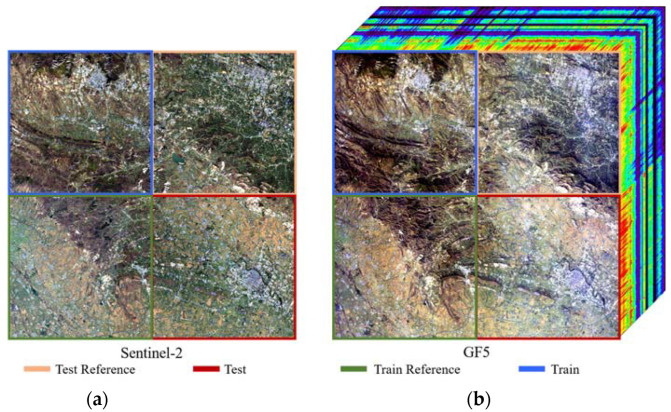
Illustration of Sentinel-2 and GF5 data sets, (**a**) is the Sentinel-2 dataset, (**b**) is the GF-5 dataset.

**Real data**. For this study, we also verified the proposed method on real data, for which Sentinel-2 and GF5 data were used. The GF5 data were acquired by the GF5-Advanced Hyperspectral Imager (AHSI) on 10 November 2019 over Xuchang city, Henan province, China. It has the size of 2083 × 2008 pixels, with 330 bands covering the spectral range 390–2500 nm and 30 m GSD. The Sentinel-2 data were acquired on 14 November 2019. We used ENVI to select the same region as the GF5 data. After image registration, each image was divided into four parts, as shown in Figure 6.

**Experimental environment.** The proposed method was implemented using the PyTorch framework [44] and trained using the Adam optimizer, which was set to its default parameters [45]. The initial learning rate was set to 3 × 10^−4^, with linear stepping decay set from epochs 500 to 2000. The parameters of the network were initialized using the Kaiming initialization method. We used randomly selected patches to train the network, and the patch size was set to 64 × 64. In the SRM, eight RDCA blocks were stacked for each backbone sub-network, and the number of features for each convolutional layer was set to 128, except for the initial convolutional layer and the last convolutional layer related to input and output bands. The training environment consisted of an Intel i-7 6850K CPU, 128 GB RAM, and 4 × GTX 1080 Ti.

**Quality assessment.** Several widely used image quality indices were used to compare the performance and reconstruction quality, including erreur relative global adimensionnelle de synthèse (ERGAS), the mean spectral angle mapper (SAM), mean peak signal-to-noise ratio (mPSNR), mean square error (MSE), and mean relative absolute error (MRAE) [11].

### 4.2. Ablation Study

To verify the effects of different modules, we implemented an ablation study on the Pavia University data set. The proposed method was composed of an STM and SRM, where the SRM is the main spectral reconstruction module and the STM is the transformer module, used to transfer the learned spectral information. We first tested the performance of the proposed network with the STM removed. Then, the hard and soft attention mechanisms were compared in order to investigate the attention behavior effect. The performance comparison, shown in Table 1, indicates that the results of the baseline SRM reached a preliminary reconstruction result. We further appended the STM modules to verify the performance under the use of hard and soft attention. We found that the accuracy of the reconstructed image gradually improved with an increase in use of the attention module. Compared with the baseline results, the hard and soft attention mechanisms demonstrated the effectiveness of the proposed learning transfer spectral structure.

### 4.3. Comparison Experiment of Simulation Data

We evaluated the performance of the proposed method with several baseline SSR methods on the simulation and real data. To investigate the performance fully, one traditional method and four CNN-based methods were used for comparison: Arad [19], AWAN [46], HRNet [47], FMNSSR [26], MSSR [27], and StiNet [24]. The parameters for all of the methods were tuned to their optimal values.

**Figure 7:**
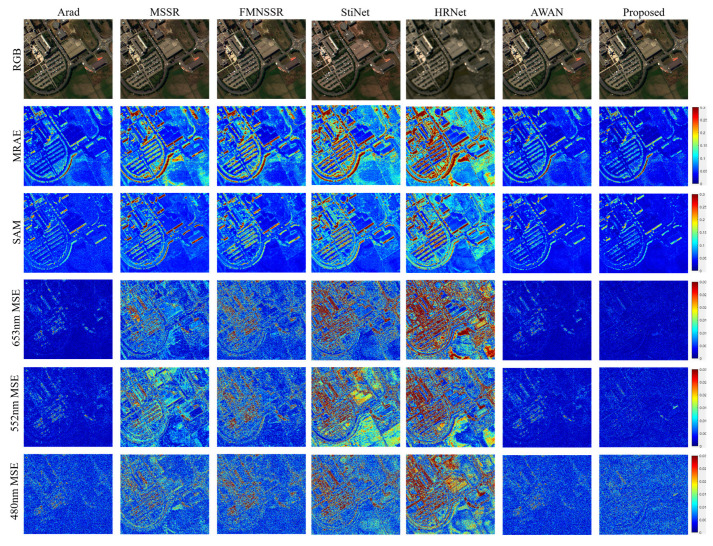
Visual comparison of the results for different methods on the Pavia University data set.

**Pavia University data**. First, the visual quality of the compared results was obtained for the Pavia University data. Figure 7 shows the reconstruction results under different indicators. The first row of Figure 7 shows the RGB images of different reconstruction results. It was found that, except for HRNet, the algorithms did not show any particular difference in the reconstruction of RGB images. Therefore, the error colormap was used to depict the reconstruction effect of different methods. The second and third rows represent the MRAE and SAM errors of the reconstruction results, respectively. The last three rows show the MSE errors in the different bands of the reconstructed image. The results demonstrate that HRNet and StiNet produced serious object boundary errors. Material-dependent errors were present in the results of MSSR and FMNSSR. In comparison, the results of Arad, AWAN, and the proposed method had fewer errors.

Table 2 summarizes the quality assessment comparison obtained when using the Pavia University data. The mPSNR indicator represents the reconstruction quality of spatial information, while the SAM indicates the spectral reconstruction quality. A similar trend to that observed in the visual comparison can be seen in the quality assessment comparison. HRNet and StiNet had the worst reconstruction quality, while MSSR and FMNSSR had a similar reconstruction quality. Arad, AWAN, and the proposed method showed stable, high-quality reconstruction results.

**Figure 8:**
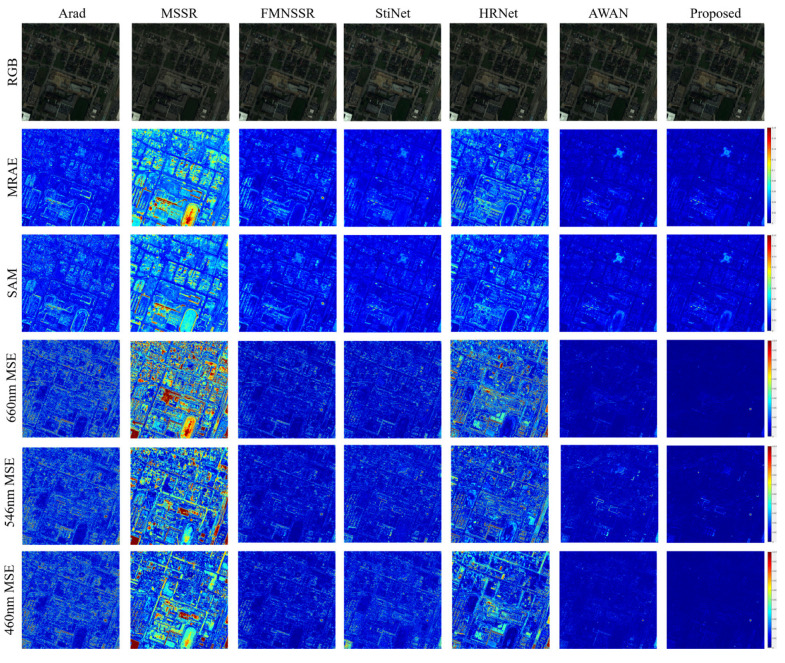
Visual comparison of the results for different methods on the University of Houston data set.

**University of Houston data**. The visual quality comparison for the University of Houston data is shown in Figure 8. Compared with the Pavia University data, the reconstruction result obtained using MSSR suffered the worst performance. This indicated that the performance of MSSR was affected by the sensors and the ground object materials. In contrast, the results of HRNet and FMNSSR showed better performances for these data. Compared with the Pavia University data, StiNet has a better reconstruction result on these data. The visual result of Arad presented the edge error for the ground object. AWAN and the proposed method yielded the best visualization reconstruction results. In contrast, the single-band comparison in the last three rows indicates that the proposed method achieved slightly better visualization results.

As presented in Table 3, the quantitative results were similar to those of the visual quality comparison. Compared to the Pavia University data, the MSSR had poor adaptability to the University of Houston data. The reason for this is that its multi-scale feature was mainly used in a way developed for spatial characteristics, while lacking the investigation of spectral information. Compared with the Arad method, FMNSSR and StiNet achieved better results in the spectral information (SAM), while the spatial reconstruction (PSNR) was not as good as that of the Arad method, which achieved stable performance in both simulated data sets, but had difficulty applying the learned dictionary to real data (the details of which are presented below). Similar to the Pavia University data, both AWAN and the proposed method delivered promising performances, but the proposed method had slightly higher accuracy.

### 4.4. Comparison Experiment on Real Data

**Figure 9:**
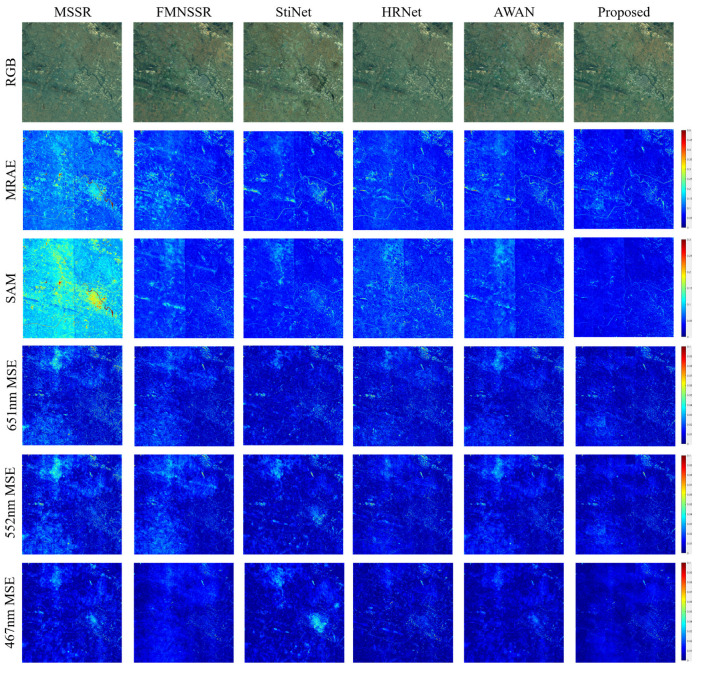
Visual comparison of the results for different methods on the GF5 data.

We also evaluated the performance of the different methods on real data. The Arad method achieved suitable performance on the simulated data, but the target image could not be reconstructed when considering real data. This was mainly because the learning method of its dictionary was based entirely on HSI images, and it was incapable of reconstructing an HSI from an MSI if there was a radiometric difference between them. Therefore, the comparison experiments only focused on the deep learning SSR methods. As shown in Figure 9, the reconstruction results from the different methods had large variability. The MSSR had poor reconstruction performance for both spatial and spectral information. In particular, there was a large distortion between the reconstructed spectral information and the real spectrum. The reconstruction results of FMNSSR, StiNet, HRNet, and AWAN were similar: they all exhibited material-dependent and significant banding errors, which were especially evident in the MRAE and SAM error images. In contrast, there were no obvious banding errors in single-band reconstruction at 467, 522, and 651 nm. With the spectral transformer as a guide, the performance of the proposed method in spectral reconstruction was promising.

Table 4 shows the reconstruction metrics for different methods. It is worth noting that the mPSNR of these data was lower than that for the simulated imagery. The main reasons were the relatively low spatial resolution of the GF5 image and the presence of a certain degree of noise in the first and last bands of the true value image. The low resolution of the image made the edges of the feature unclear, and the noise in the original image of GF5 made it so that the accuracy calculation did not reflect the real reconstruction effect. Nevertheless, the method proposed in this paper achieved optimal results in the reconstruction index.

**Figure 10:**
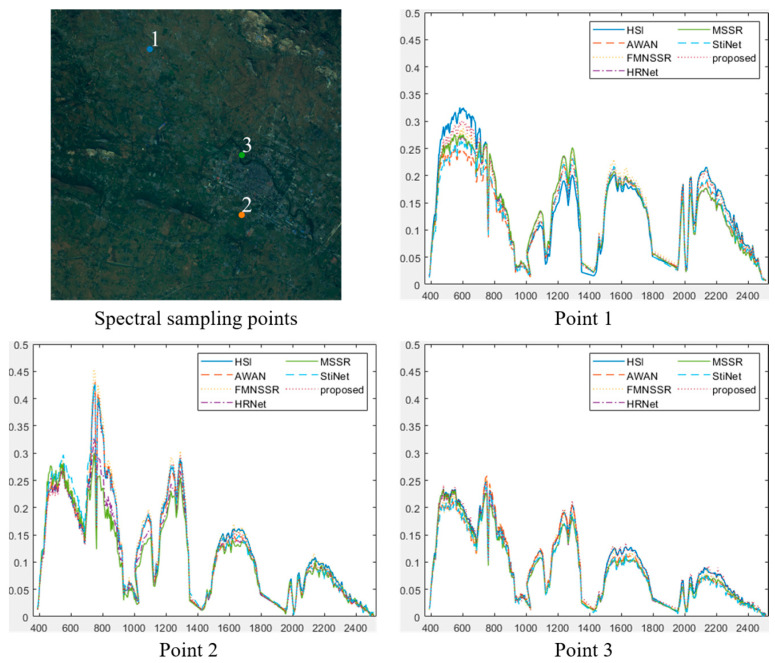
Spectral curve comparison at selected points in the GF5 data, showing the results for the various methods.

In addition, we plotted the spectral curves to compare the spectral reconstruction effect of different SSR methods, as shown in Figure 10. Point 1 represents the comparison of spectral curves in bare soil areas, while points 2 and 3 indicate different types of vegetation. Compared with the other methods, the spectral curve of the proposed method was closer to the actual spectral curve. The main reason for this was the use of transfer learning in the proposed method, which migrated the spectral information from the reference image to obtain a more accurate reconstruction result.

### 4.5. Comparison Time Complexity

In addition, we further compared the time complexity of the different methods, as shown in Table 5. It can be found that a more complex model corresponds to a higher time complexity. Although the proposed method has the lowest time efficiency, its reconstruction accuracy is relatively the best.

## 5. Conclusions

In this work, a novel spectral super-resolution network, called the learning spectral transformer network (LSTNet), was proposed, in which the spectral structure of a reference HSI is utilized through transfer learning to promote a reasonable reconstruction spectrum. The main contributions of the work can be summarized as follows:

1. The proposed method is composed of an SRM (spectral reconstruction module) and an STM (spectral transformer module) to learn the prior spectral information and reference spectrum.
2. The STM (spectral transformer module) is used to compute the spatial relevance between the input MSI and reference MSI, and the related spectral structures are transferred from the reference HSI into the spectral reconstruction network.
3. In the SRM (spectral reconstruction module), the channel attention mechanism is used to enhance the channel-wise adaptive learning, through which the model can effectively reallocate weights along the spectral dimension. The proposed network is capable of transferring the spectral information from the reference image and adaptively adjusting the weights of the features.

Extensive experiments conducted on two simulated benchmark data sets and a real data pair demonstrated the effectiveness of the proposed method compared to state-of-the-art methods, along with its ability to produce highly accurate reconstructed images.

**Challenges and Trends**: Due to the limitations of the imaging system, a hyperspectral imaging system usually needs to make trade-offs in field of view, focal length, and imaging CCD size to meet the high imaging signal-to-noise ratio. This makes it difficult for the hyperspectral images acquired by the current satellite to simultaneously meet the usage requirements of wide swath and high spatial resolution. Enormous efforts have been made to develop hyperspectral image reconstruction techniques, such as spatio-spectral fusion, single image super-resolution, and spectral super-resolution. The major remaining issue in the field of spatio-spectral fusion is the development of more accurate fusion methods for the inconsistency between coarse and fine spatial resolution images caused by the atmospheric conditions or the acquisition geometry. The challenge of single-image super-resolution reconstruction is the effectiveness of spatial information reconstruction at larger scaling factors rather than simply sharpening ground object edges. The main challenge for spectral super-resolution reconstruction is to develop reconstruction methods with more spectral fidelity and interpretability.

## Figures and Tables

**Table 1:** Ablation study on the Pavia University data set.

Component Modules			Accuracy Indicators	
STM	Hard Attention	Soft Attention	mPSNR	SAM
✓	✗	✗	40.6894	3.6826
✓	✓	✗	41.2751	3.4154
✓	✗	✓	41.4929	3.2163
✓	✓	✓	**42.8197** ^1^	**2.1273** ^1^

^1^ The best results are shown in bold. ✓ represents yes, ✗ represents no.

**Table 2:** Quality assessment comparison on the Pavia University data set.

	Arad	MSSR	FMNSSR	StiNet	HRNet	AWAN	Proposed
mPSNR	41.3532	36.0617	36.1042	31.3055	29.1888	42.2823	**42.8197** ^1^
SAM	3.1857	4.2996	3.9235	5.3270	6.1036	2.3701	**2.1273** ^1^
ERGAS	8.1241	11.5657	11.4860	19.0757	23.3450	6.5043	**6.3161** ^1^
RMSE	0.0121	0.0163	0.0161	0.277	0.0354	**0.0088** ^1^	**0.0088** ^1^

^1^ The best results are shown in bold.

**Table 3:** Quality assessment comparison on the University of Houston data set.

	Arad	MSSR	FMNSSR	StiNet	HRNet	AWAN	Proposed
mPSNR	51.2520	41.5942	49.4653	50.0773	44.5608	55.4147	**55.4916** ^1^
SAM	1.9206	3.0962	1.4491	1.1656	1.91728	0.9282	**0.9107** ^1^
ERGAS	5.0095	7.9960	3.8359	3.244	5.4504	2.4631	**2.3767** ^1^
RMSE	0.0046	0.0087	0.0036	0.003	0.0062	**0.0024** ^1^	**0.0024** ^1^

^1^ The best results are shown in bold.

**Table 4:** Quality assessment comparison on GF5 data.

	MSSR	FMNSSR	StiNet	HRNet	AWAN	Proposed
mPSNR	27.9931	27.7670	27.8081	28.0959	28.8122	**29.3163** ^1^
SAM	4.3814	3.8177	3.9325	4.1560	3.70281	**3.0861** ^1^
ERGAS	15.0157	15.1137	15.2949	15.0880	13.1582	**12.8030** ^1^
RMSE	0.0189	0.0187	0.0192	0.0184	0.0167	**0.0151** ^1^

^1^ The best results are shown in bold.

**Table 5:** Time complexity comparison for different methods (unit: second).

	Arad	MSSR	FMNSSR	StiNet	HRNet	AWAN	Proposed
Pavia	2.147	**0.2608** ^1^	0.3453	0.5831	0.7218	1.0181	0.84173
IGARSS	7.195	**0.8354** ^1^	1.0996	1.7469	2.2321	5.276	4.8896
GF5	66.705	**3.29** ^1^	4.8014	5.383	5.8663	13.8558	15.2629

^1^ The best results are shown in bold.

## Data Availability

Not applicable.
